# Pathogenic expansion: fibroblast proliferation fuels fibrosis

**DOI:** 10.1172/JCI199180

**Published:** 2025-11-17

**Authors:** Cody A. Schott, Elizabeth F. Redente

**Affiliations:** 1 Division of Pulmonary Sciences and Critical Care Medicine, Department of Medicine, University of Colorado, Aurora, Colorado, USA.; 2 Department of Pediatrics, National Jewish Health, Denver, Colorado, USA.

## Abstract

Pulmonary fibrosis, an unrelenting disease of lung scarring, has been associated with the expansion of a profibrotic fibroblast population and extensive extracellular matrix deposition. In this issue, Molina and colleagues provide foundational mechanistic evidence that fibroblast proliferation itself is a critical driver of fibrosis. Using lineage tracing in preclinical fibrosis models, the authors showed that naive *Scube2^+^* alveolar fibroblasts underwent a profibrotic phenotypic switch prior to proliferating within areas of fibrotic remodeling. Induction of apoptosis via *Esco2* deletion or directly preventing proliferation via *Ect2* deletion in these fibroblasts attenuated fibrosis. Complementary analyses on explanted human lung tissue confirmed translational relevance, collectively providing compelling evidence for the importance of fibroblast proliferation in fibrotic disease.

## Preclinical evidence of pathogenic fibroblast proliferation driving fibrosis

Fibroblasts and their extracellular matrix (ECM) products have long been recognized as mediators of pulmonary fibrosis. They are a heterogeneous cell type involved in architectural maintenance, fibrosis, and inflammatory signaling (1, 2). Preclinical studies of pulmonary fibrosis utilize endpoints of fibroblast biology and collagen synthesis as critical in vivo and in vitro surrogates to understand human disease activity. However, important questions about temporal expansion and acquisition of a profibrotic phenotype in fibroblasts have remained unanswered: Which occurs first, and are both processes necessary and/or sufficient for fibrosis development? In this issue of the *JCI*, Molina et al. implicated alveolar fibroblast proliferation after profibrotic differentiation as an essential step in the development of pulmonary fibrosis (3).

**Figure 1:**
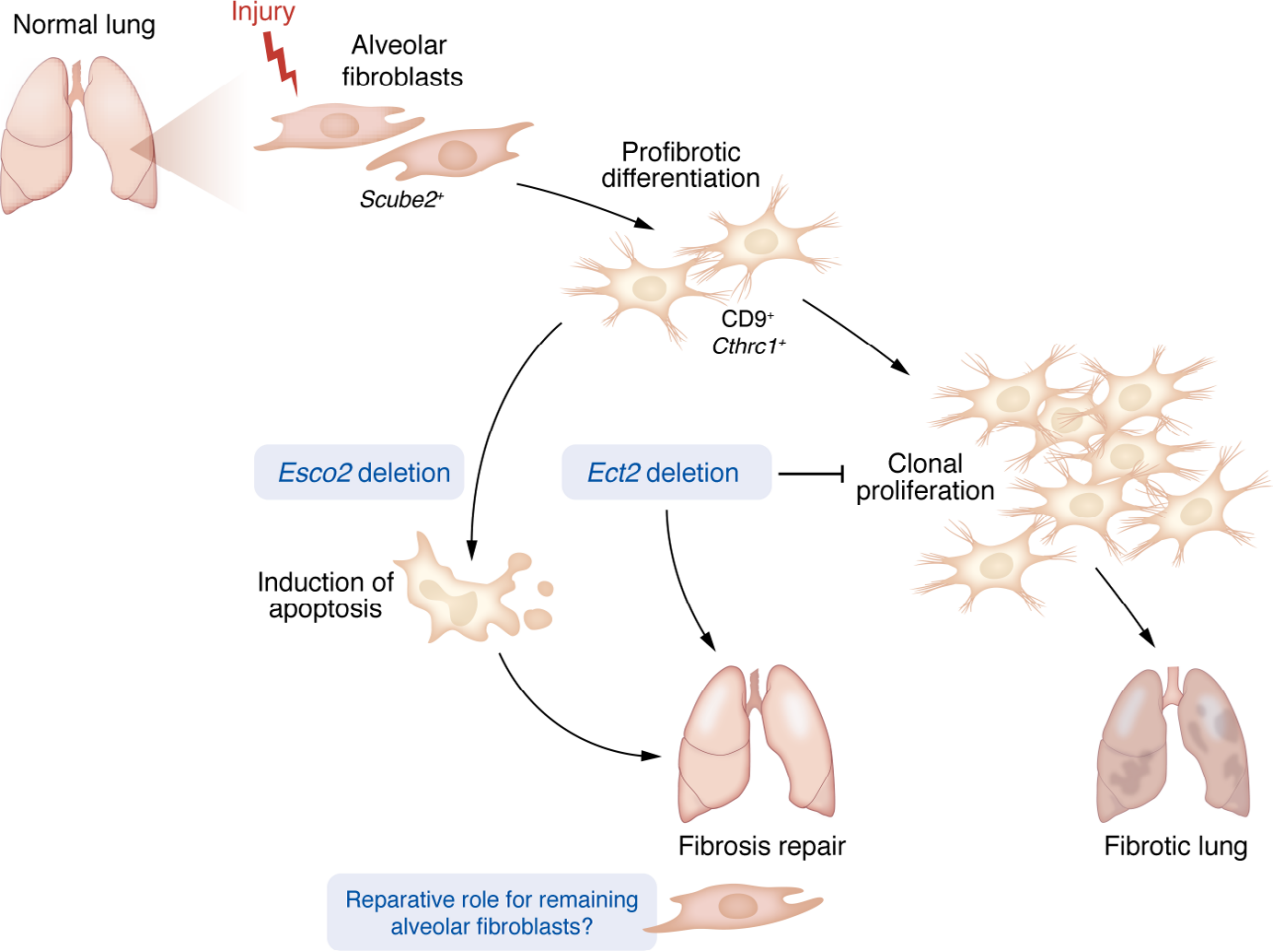
Schematic of fibroblast response to injury during fibrosis development. Molina et al. demonstrated that alveolar fibroblasts, defined by *Scube2* expression, first undergo profibrotic differentiation into a CD9*^+^*, *Cthrc1^+^* population. Differentiation into this population was followed by clonal proliferative expansion. Genetic deletion of either *Esco2*, which induces apoptosis in the profibrotic population, or *Ect2*, which inhibits clonal proliferation of the population, reduced proliferating fibroblasts and fibrosis development (3).

While previous studies have demonstrated that a quantitative increase in fibroblast numbers correlates with fibrosis development (2, 4–6), Molina and colleagues extended these observations, providing evidence that *Scube2^+^* fibroblasts, which this group recently characterized as the naive alveolar fibroblast capable of generating multiple fibroblast subsets (2), gained CD9 expression and a fibrotic transcriptomic profile (*Cthrc1^+^*, *Col1a1*, *Tnc^+^*, etc.) prior to proliferation in two distinct murine fibrosis models (single-dose bleomycin and silica) Using *Scube2-CreER Brainbow2.1/+* confetti mice and 5-ethynyl-2′-deoxyuridine (EdU) incorporation, clonal expansion of fibroblasts was observed within regions of fibrotic remodeling (3). To determine whether this subpopulation functionally contributes to the development of fibrosis, the proliferative process was disrupted in two ways. First, by deleting *Esco2* in proliferating *Scube2^+^* fibroblasts, the chromatin cohesion complex was targeted to induce apoptotic cell death during fibrogenesis, which resulted in a significant reduction in pulmonary fibrosis development (Figure 1) (3). Previous studies in which apoptosis of collagen-expressing fibroblasts was inhibited through the genetic loss of *Fas* demonstrated a complementary result and sustained fibrosis in the bleomycin model (6). Second, to disrupt proliferation without cell death, *Ect2* (which impairs cytokinesis) was deleted in *Scube2^+^* alveolar fibroblasts. This resulted in significantly fewer fibroblast aggregates and an overall reduction in fibrosis development (Figure 1) (3).

Because not all of the *Scube2^+^* subpopulation differentiated during fibrosis, there remained a nonproliferating fibroblast population in the lungs. Despite this, an improvement in fibrosis occurred, indicating that resident fibroblasts that do not undergo differentiation and that subsequent proliferation may also have a distinct role in directing the repair process (Figure 1) (7, 8). This reparative capacity has been well studied in other organ fibrosis including in the liver, kidneys, and skin (9, 10). These results suggests that a threshold must be exceeded by profibrotic fibroblasts through proliferation (or eventually resistance to apoptosis) to drive aberrant fibrotic repair, and that the presence of a small number of profibrotic fibroblasts themselves is not sufficient to overcome a homeostatic repair process (3, 6).

In usual interstitial pneumonia (UIP) associated with idiopathic pulmonary fibrosis (IPF), there is spatial, temporal, and transcriptomic heterogeneity of fibroblastic foci and profibrotic fibroblasts found within the lungs (1, 11, 12). However, within the fibroblastic foci themselves there is evidence of a paucity of fibroblast proliferation (1, 13). This implies that adjacent fibrotic areas of the lung with ongoing profibrotic activity and fibroproliferation may contribute to the progressive nature of the disease (11). Crosstalk between aberrant epithelial cell populations (alveolar cells, transitional cells, aberrant basaloid cells, airway cells, and those in honeycomb cysts) and resident and recruited immune cells may also contribute to the signaling that drives the proliferation of *Cthrc1^+^* fibroblasts, a profibrotic subpopulation derived from *Scube2^+^* fibroblasts (14–17). As an example, in the bleomycin model, *Krt8^+^* transitional epithelial cell development is tied to macrophage recruitment and fibroblast persistence, and its genetic loss is sufficient to prevent fibrosis development (18). Further investigation into the interdependent signaling loops between the *Krt8^+^* transitional epithelial cells and profibrotic fibroblasts is warranted to determine whether a signaling interaction between these cell populations drives differentiation and proliferation.

## Addressing profibrotic fibroblast proliferation in human fibrosis

The current antifibrotic therapeutics, nintedanib and pirfenidone, target fibroblast activation, differentiation, ECM production, and proliferation. This suggests that the design of additional targeted fibroblast depletion therapies may be valuable for the treatment of pulmonary fibrosis (19–21). To examine fibroblast proliferation in human lungs and validate their preclinical findings, Molina et al. utilized precision-cut lung slices (PCLSs) from healthy lungs and from explants from patients with IPF and silicosis. Similar increases in fibroblast proliferative capacity were observed in profibrotic fibroblasts isolated from IPF and silicotic human lungs compared with healthy human lungs (3). This supports the concept that fibroblast proliferation remains an ongoing process as part of progressive disease pathogenesis in humans. However, while the PCLS model is an important and valid one for analyzing human lung tissue, a few caveats should be noted. Analyses with PCLSs are frequently limited by sample acquisition (in this case only 1 silicosis lung was available), often only represent late-stage disease, have variability in the tissue region that is isolated, lack infiltrating immune cells, and have poor long-term viability in culture. In the PCLS model, treatment of normal lungs with a profibrotic cocktail initiates limited fibrotic changes; however, this system may also provide additional insight into the early events that trigger the profibrotic switch and subsequent proliferative response (22).

Preclinical models have a limited ability to reproduce the chronicity of human disease, making it difficult to predict which pathways maintain clinical significance for successful therapeutic development (23). In the single-dose bleomycin model, the waves of fibroblast proliferation and pathogenic subpopulation expansion are self-limiting, with spontaneous apoptosis followed by fibrosis resolution occurring by 9 weeks (3, 6, 24). Models using silica (as used here by Molina et al.) or repetitive dosing of bleomycin better reflect the persistent disease that occurs in humans and can be utilized to study the progression from acute to chronic processes (3–5). Evaluating the role and extent of fibroblast proliferation in the chronic phases of these models could, as suggested by the PCLS experiments, identify the mechanisms underlying how fibroblast proliferation remains an active pathogenic process in progressive fibrosis, and whether crosstalk with aberrant epithelial cell populations and immune cells helps to maintain a proliferative pathogenic fibroblast pool. Ultimately, a better understanding of chronic in vivo fibroblast proliferation could identify a primary clinical therapeutic target for pulmonary fibrosis.

Clinically, these findings suggest that a shift in the therapeutic focus toward targeting the proliferative capacity of profibrotic fibroblasts rather than broadly suppressing fibroblast function may be a viable direction. Recent studies have also suggested that targeting the antiapoptotic protein signature of fibroblasts with BH3 mimetics also has therapeutic potential (4). Additional strategies may include using inhibitors of cell-cycle regulators, cytokinesis pathways, or proliferation-specific kinases. Furthermore, the discovery that profibrotic fibroblasts proliferate after acquiring a pathogenic phenotype implies that therapeutic windows may extend into established disease, offering opportunities for intervention even after fibrosis is underway. This could be particularly valuable in IPF, where late-stage diagnosis is common.

## Conclusions

Molina and colleagues have identified a new pathogenic paradigm in pulmonary fibrosis, whereby alveolar fibroblasts undergo a profibrotic phenotypic switch prior to proliferative expansion. While previous studies were inconclusive as to the degree and importance of fibroblast proliferation in fibrosis, this study showed that (a) profibrotic fibroblasts proliferate after differentiation, (b) they are located within collagen-dense tissue regions, and (c) inhibition of proliferation or targeted apoptotic loss of this population meaningfully reduces clinical evidence of fibrosis (Figure 1). The elevated proliferative capacity of fibroblasts observed in human fibrotic lungs further underscores the clinical utility of therapeutically targeting fibroblast proliferation. Additionally, we note that the authors reanalyzed published single-cell RNA-Seq data, which serves as a reminder that as scientific context and paradigms evolve, the use of existing sequencing data remains a powerful tool to better characterize and validate findings from novel experiments (12).

This work helps to reframe the role of fibroblast proliferation from an epiphenomenon to a central driver of pulmonary fibrosis development. Prior literature contains conflicting reports, often with the in vitro cofounders of variable culture conditions and cellular heterogeneity. One of the biggest assumptions has been that fibroblasts proliferate prior to differentiation and the acquisition of a pathogenic profibrotic phenotype. However, the studies presented in the work by Molina et al. showed that a subset of *Scube2^+^* alveolar fibroblasts first acquired a CD9*^+^* *Cthrc1^+^* profibrotic phenotype, followed by clonal expansion, establishing fibroblast proliferation as a vital step for disease progression. This mechanistic study integrated heterogeneity, spatial biology, and functional causality, redefining fibroblast proliferation as a cornerstone of fibrotic lung disease pathogenesis.

## Funding support

This work is the result of NIH funding, in whole or in part, and is subject to the NIH Public Access Policy. Through acceptance of this federal funding, the NIH has been given a right to make the work publicly available in PubMed Central.

NIH grant T32HL007085 (to CAS).
NIH grant R01HL166250 (to EFR).

## Version 1 11/17/2025

Electronic publication
